# Factors associated with Severe Fever with Thrombocytopenia Syndrome infection and fatal outcome

**DOI:** 10.1038/srep33175

**Published:** 2016-09-08

**Authors:** Jimin Sun, Zhenyu Gong, Feng Ling, Rong Zhang, Zhendong Tong, Yue Chang, Enfu Chen, Qiyong Liu, Junfen Lin, Zhiping Chen, Jianmin Jiang

**Affiliations:** 1 Zhejiang Provincial Center for Disease Control and Prevention, Hangzhou, China; 2 State Key Laboratory of Infectious Disease Prevention and Control, Collaborative Innovation Center for Diagnosis and Treatment of Infectious Diseases, National Institute for Communicable Disease Control and Prevention, Chinese Center for Disease Control and Prevention, Beijing, China; 3 Zhoushan Municipal Center for Disease Control and Prevention, Zhoushan, China; 4 Taizhou Municipal Center for Disease Control and Prevention, Taizhou, China

## Abstract

Severe fever with thrombocytopenia syndrome (SFTS) is emerging in China and the incidence increased year by year. In this study, we conducted case control study to explore factors associated with SFTS virus (SFTSV) infection and fatal outcome. In the study of factors associated with SFTSV infection, a total of 216 individuals participated the study, including 72 cases and 144 matched controls. There were significant differences in proportion of history of tick bite and breeding domestic animals between cases and controls. Of note, individuals who were unclear whether they had been bitten by ticks had the highest risk of SFTSV infection and odds ratio (OR) was 10.222. In the study of factors associated with SFTS fatal outcome, a total of 129 cases participated the study including 16 deaths and 113 survivors. Significant differences were observed in body mass index (BMI), intervals from illness onset to confirmation, and proportion of gingival hemorrhage between deaths and survivors, whose ORs of these factors were 3.903, 1.996, and 3.826, respectively. Our results suggest that all patients with fever, thrombocytopenia and leukocytopenia in SFTS endemic areas should be suspected of SFTS, even they don’t have history of tick bite, and more intense treatment should be administered to patients with abnormal BMI before laboratory parameters are detected.

Severe fever with thrombocytopenia syndrome (SFTS) is an emerging infectious disease which is caused by SFTS virus (SFTSV), a novel member of the *Phlebovirus* genus in the *Bunyaviridae* family^1^,^2^. The clinical symptoms of SFTS include fever, fatigue, chill, headache, lymphadenopathy, anorexia, nausea, myalgia, diarrhea, vomiting, abdominal pain, gingival hemorrhage, conjunctival congestion, and so on^3^. Notably, SFTS patients have an extensively wide clinical spectrum, with some experiencing self-limiting clinical course, while approximately 12% of the cases developing fatal outcome^4^,^5^. SFTS was first reported in the rural areas of Hubei and Henan provinces in Central China in 2009^1^. As of 2016, SFTS like or confirmed SFTS patients have been reported in South Korea, Japan, United Arab Emirates, and United Stated outside China^6^,^7^,^8^,^9^.

SFTSV is believed to be transmitted through tick bites, direct contact with SFTS patients’ blood or secretion, and probable aerosol transmission^10^,^11^,^12^,^13^,^14^,^15^. Identification of risk factors is of vital importance for the control and prevention of SFTSV infection. In 2011, a study on risk factors for SFTSV infection was conducted in Henan Province, Hubei Province, and Shandong Province. They reported that farmers were more common among cases and tick bites, cat or cattle ownership and presence of weeds and shrubs in the working environment were risk factors^16^. However, all controls were selected from hospitals and they might have not been drawn from the same environment in that study^16^. These might lead to overpower factors including occupation, tick bites, and habitats around residence places associated with environment. Moreover, many people lived in similar environments with cases and why other people were not infected with SFTSV? In order to explore further risk factors for SFTSV infection, we selected controls from villages where cases occurred in our study.

Several previous studies reported that some laboratory parameters and clinical symptoms were associated with SFTS fatal outcome. Liu W. *et al*. reported that decreased level of consciousness, and elevated levels of lactate dehydrogenase and creatine kinase were significantly associated with fatality of SFTS cases^17^. Sun Y. *et al*. confirmed that Cytokines IL-1RA, IL-6, IL-10, G-CSF, IP-10, and MCP-1 were elevated in SFTS patients and produced at robust levels in fatal cases^18^. Fatal outcome of SFTS cases was also believed to be associated with high viral RNA load in blood at admission, higher serum liver transaminase levels, more pronounced coagulation disturbances, and higher levels of acute phase proteins, cytokines, and chemokines^19^. Similarity, Gai Z. T. *et al*. reported that a period of 7–13 days after the onset of illness was the critical stage and the key risk factors that contributed to patient death were elevated serum aspartate aminotransferase, lactate dehydrogenase, creatine kinase, creatine kinase fraction, the appearance of central nervous system (CNS) symptoms, hemorrhagic manifestation, disseminated intravascular coagulation, and multi-organ failure^20^. However, it might be too late to cure SFTS cases when some laboratory parameters are detectable and some clinical symptoms occur. In this study, not only clinical symptoms associated with fatal outcome were analyzed but also other risk factors which can be identified when cases were admitted were also analyzed.

## Results

### Factors associated with SFTSV infection

In the case control study of factors associated with SFTSV infection, a total of 216 individuals including 72 cases and 144 matched controls participated the study in 2015. The median age of cases was 64.5 years (interquartile range, 57–74.75 years) and the median age of controls was 65 years (interquartile range, 56.25–72 years). There was no significant difference in age between cases and controls (P = 0.372 > 0.05).

As shown in Table 1, variables including underlying condition, Body Mass Index (BMI), outdoor activity, history of tick bite, breeding domestic animal, rodents around habitat, grasses around habitat were assigned in the single variable and multivariable logistic regression analysis. According to results of single variable logistic regression analysis, no significant differences in underlying condition, BMI, outdoor activity, rodents around habitat, and grasses around habitat were observed between cases and controls (Table 2). However, there were significant differences in history of tick bite and breeding domestic animals between cases and controls. Odds ratio (OR) of breeding domestic animals was 1.834 (95% CI: 1.030–3.264). To our surprise, individuals who were unclear whether they had been bitten by ticks had the highest risk of SFTSV infection and OR was 10.222 (95% CI: 4.265–24.50). According to results of multivariable logistic regression analysis, history of tick bite and breeding domestic animal entered final equation. Wals of these two variables were 20.469 and 20.229, respectively and OR were 6.592 (95% CI: 2.892–14.994) and 1.745 (95% CI: 1.000–3.045), respectively.

### Factors associated with SFTS fatal outcome

In the case control study of factors associated with SFTS fatal outcome, a total of 129 cases including 16 deaths and 113 survivors participated the study during 2014 and 2015. There were 7 male and 9 female in death group and 39 male and 74 female in survivor group. No significant difference was observed in gender distribution between the two groups (P = 0.470 > 0.05). The median age of deaths was 67 years (interquartile range, 61.25–72.5 years) and the median age of survivors was 63 years (interquartile range, 55–72 years). There was no significant difference in age between deaths and survivors (P = 0.188 > 0.05).

Variables including underlying condition, BMI, interval from illness onset to confirmation, and some clinical symptoms (Chill, headache, fatigue, muscular soreness, conjunctival congestion, petechiae, gingival hemorrhage, anorexia, nausea, vomiting, haematemesis, abdominal pain, abdominal distension, diarrhea, lymphadenopathy) were assigned in the single variable and multivariable logistic regression analysis (Table 3). The median interval from illness to confirmation for deaths and survivors was 7 days, and 5 days, respectively. According to results of single variable logistic regression analysis, there were significant differences in BMI, interval from illness onset to confirmation, and gingival hemorrhage between deaths and survivors and other factors were similar between the two groups. OR of BMI, interval from illness onset to confirmation, and gingival hemorrhage were 3.903 (95% CI: 1.331–11.449), 1.996 (95% CI: 1.162–3.428), and 3.826 (95% CI: 1.136–12.891), respectively (Table 4). According to results of multivariable logistic regression analysis, variables in the equation included BMI and interval from illness onset to confirmation. Wals of the two variables were 5.702 and 5.911, respectively and OR were 3.886 (95% CI: 1.275–11.84) and 1.956 (95% CI: 1.139–3.361), respectively.

## Discussion

SFTSV has been detected in ticks from China, South Korea, and Japan in recent years^21^,^22^,^23^,^24^. Tick bite was identified as a risk factor for SFTSV infection in a previous study^16^. However, tick bite was not more common among cases than controls and more cases were unclear whether they had been bitten by ticks in our study. The reasons may be that tick bite are commonly painless, some patients aren’t familiar with ticks. Hence, they don’t know whether they have been bitten by ticks. Even they have bitten by an arthropod, they don’t know whether that it is tick or not. These results inform that not only persons with history of tick bite but also persons who are unclear whether they have been bitten by ticks have high risk of SFTSV infection. In addition, relationship between SFTSV infection and tick species, duration of tick attachment, different life stage (larva, nymph, adult) and the way of removing ticks need further research.

Similar to results of a previous study, breeding domestic animals including dogs, cattle, goats, and chickens was another risk factor for SFTSV infection^25^. The data indicate that these domestic animals may be potential reservoir hosts of SFTSV. Some studies also confirmed that SFTSV-specific antibodies were detected in specimens from sheep, cattle, dogs, pigs, and chickens^26^,^27^,^28^. As these domestic animals were host of some ticks, breeding domestic animals may lead to an increase of tick bite which increase probability of SFTSV infection. Additionally, cases might also be infected with SFTSV via contact with secretions of animals although this may not be the major transmission route.

Ages of most SFTS cases ranged between 50 and 74 years and age was believed to be a critical risk factor for SFTS^29^,^30^. But there was no significant difference in age distribution among two groups as age was matched to increase statistical power and identify other risk factors in our case control study. To our disappointment, underlying condition and BMI were also not more common in cases. The reasons may be that age was matched in our study and underlying condition were related to age.

Different with results of previous studies, there were no significant differences in rodents around habitat and grasses around habitat between cases and controls. These results can be attributed to the design of the study. Controls were selected from the same village of cases and they lived in similar environments which may underestimate risk factors associated with environment.

Contrary to results of another study that older age is a risk factor for SFTS fatal outcome, age distribution was similar between deaths and survivors in our case control study of SFTS fatal outcome^16^. These may be relative with age distribution of all subjects and sample size. All subjects in our study were from Zhejiang Province and most of them aged from 50 to 74 years. Furthermore, only 16 deaths occurred in Zhejiang Province during 2014 and 2015. Small sample size may lead to bias and underestimate OR of age. However, age might be a façade for SFTS fatal outcome. Some factors associated with age may be really relative to SFTS fatal outcome. Although no significant difference in underlying condition was observed between deaths and survivors, OR of underlying condition was 2.927.

BMI is a measure of relative size based on the mass and height of an individual. It is used as a screening tool to indicate whether a person is underweight, overweight, obese or a healthy weight for their height. In our study, cases whose BMI were out of the healthy range were more likely to die than cases whose BMI were in the healthy range. Given that no existing literature has discussed this topic, we hypothesize one possible explanation. BMI represent body condition of persons and normal BMI means better immunological status. Nevertheless, our study demonstrated that BMI was a useful predictor of fatal outcome suggesting more attention should be paid to SFTS cases whose BMI < 18.5 or >24 during treatment.

Early diagnosis is very important for treatments of SFTS cases. Our study indicates that an increase in interval from illness onset to confirmation by 3 days was associated with fatality with an OR of. Longer interval from illness onset to confirmation can lead to a delay in the key period for treatment. The delay in confirmation may be related to poor capacity of SFTS identification in most hospitals. The majority of hospitals don’t have the capacity to detect SFTSV and most samples are transported to municipal centers for disease control and prevention (CDC) or provincial CDC for testing. Our funding further suggests that hospitals in SFTS endemic areas should improve capacity of SFTSV detection to shorten the interval from illness onset to confirmation and decrease fatality.

In the single variable analysis of our study, gingival hemorrhage contributed to fatal outcome of SFTS cases. We tentatively speculate that gingival hemorrhage is a parameter of severity of disease and patients with gingival hemorrhage are more serious.

There are several limitations in our study. First, age was matched in our study of factors associated with SFTSV infection. Some factors associated with age (i.e., underlying condition, and BMI) might be underestimated. Second, all controls were selected from villages where SFTS patients lived. Similar habitats of cases and controls may lead to underestimation of environmental risk factors. Finally, most young adults migrate from rural areas to urban areas and rural areas are left with an age distribution skewed towards seniors in Zhejiang Province. As a result, the majority of cases aged 50–74 years and only few young adult cases were reported. Age distribution of survivors and deaths was too limited and bias may occur when we analyzed the relation between age and fatal outcome.

Despite the limitations stated above, our study identified two factors associated with SFTSV infection and three novel factors associated with fatal outcome. Different with other studies, tick bite was not more common among cases in our study. Instead, more cases were unclear whether they had been bitten by ticks. The results inform that history of tick bite is not essential for diagnosis of SFTS and all patients with fever, thrombocytopenia and leukocytopenia in areas where ticks exist should be suspected of SFTS no matter they have history of tick bite or not. To the best of our knowledge, we reported firstly that BMI and interval from illness onset to confirmation were successful predictors for SFTS fatal outcome. The data suggest that more intense treatment should be administered to patients with abnormal BMI before laboratory parameters are detected and comprehensive measures should be done to shorten interval from illness onset to confirmation.

## Methods

### Definitions of cases and controls

In accordance with the national guideline for prevention and control for SFTS issued by the Chinese Ministry of Health, an acutely ill person with acute onset of fever (≥ 38.0 °C) and other symptoms (e.g., gastrointestinal symptoms, bleeding), epidemiological risk factors (being a farmer or being exposed to ticks 2 weeks prior to illness onset), and laboratory data showing thrombocytopenia and leukocytopenia, was defined as a suspected case of SFTSV. Confirmed cases of SFTSV infection were defined as those who met the criteria for a suspected case of SFTSV and who also met one or more of the following criteria: (1) detection of SFTSV RNA by a molecular method, (2) seroconversion or 4-fold increase in antibody titers between two serum samples collected at least 2 weeks apart, and (3) isolation of SFTSV in cell culture^31^. In this study, case subjects were defined as confirmed SFTS cases. Control subjects were defined as matched persons whose laboratory testing for SFTSV infection (RT-PCR, IgM and IgG ELISA) were negative. The methods were carried out in accordance with the appendix (guideline for laboratory detection) of the national guideline for prevention and control for SFTS issued by the Chinese Ministry of Health. All experimental protocols were approved by the National Institute for Viral Disease Control and Prevention, China and some protocols were provided by them.

### Study Design

Zhejiang Province Center for Disease Control and Prevention (Zhejiang CDC) designed the case control study of factors associated with SFTSV infection and fatal outcome. In the case control study of factors associated with SFTSV infection, case group was composed of SFTS cases who were reported in the National Notifiable Disease Surveillance System (NNDSS) in 2015 in Zhejiang Province. Controls were randomly selected from individuals who lived in same villages with SFTS cases. They were matched by age (+/−5 years) and the matching ratio was 1:2.

In the case study of factors associated with SFTS fatal outcome, survivor group included all survived SFTS cases reported in 2014 and 2015 in Zhejiang Province. Death group was comprised of all dead SFTS cases reported in 2014 and 2015 in Zhejiang Province.

### Data Collection

The aims of our study were explained to all subjectsand their informed consents were obtained in this study. SFTS patients and controls were asked about their demographic features (age, gender, occupation, residential address, height, and weight), underlying conditions (diabetes and hypertension), living environment (e.g., animal raising, house rats), and exposure history (outdoor activities, tick bites). Clinical signs and symptoms, date of illness onset, and date of confirmation of SFTS diagnosis were also collected according to their medical records. Data were double entered into an Epidata 3.02 (the EpiData Association, Denmark) database followed by consistency checking.

### Data Analysis

SPSS version 20.0 (Statistical Product and Service Solutions, Chicago, IL, USA) was used for all statistical analyses. All tests were 2-tailed and statistical significance was set at P < 0.05. Fisher’s exact test or Wilcoxon Rank Sum W Test were used, as appropriate, to compare the characteristics of cases and controls, deaths and survivors. Single variable analysis and multivariate analysis were conducted to identify factors associated with SFTSV infection and fatal outcome using the logistic regression method. Moreover, OR of all factors were also calculated.

## Additional Information

**How to cite this article**: Sun, J. *et al*. Factors associated with Severe Fever with Thrombocytopenia Syndrome infection and fatal outcome. *Sci. Rep.* **6**, 33175; doi: 10.1038/srep33175 (2016).

## Figures and Tables

**Table 1:** Assignment of variables in logistic regression analysis of factors for SFTSV infection.

Variable	Assignment
Underlying condition	No = 0, Yes = 1
BMI	18.5∼24 = 0, <18.5 or >24 = 1
Outdoor activity	No = 0, Yes = 1
History of tick bite	No = 0 0, Yes = 1 0, Unclear = 0 1
Breeding domestic animal	No = 0, Yes = 1
Rodents around habitat	No = 0, Yes = 1
Grasses around habitat	No = 0, Yes = 1

**Table 2:** Single variable analysis on factors for SFTSV infection.

Variable	B	SE	Wals	P	OR	95% CI	
Underlying condition	0.128	0.309	0.172	0.679	1.136	0.621	2.081
BMI	−0.415	0.315	1.738	0.187	0.660	0.356	1.224
Outdoor activity	−0.139	0.321	0.186	0.666	0.871	0.464	1.633
History of tick bite			27.222				
Yes	0.498	0.350	2.025	0.155	1.646	0.829	3.269
Unclear	2.325	0.446	27.167	0.000	10.222	4.265	24.50
Breeding domestic animal	0.606	0.294	4.251	0.039	1.834	1.030	3.264
Rodents around habitat	−0.148	0.382	0.150	0.699	0.863	0.408	1.822
Grasses around habitats	0.387	0.499	0.602	0.438	1.472	0.554	3.912

**Table 3:** Assignment of variables in logistic regression analysis of factors for fatal outcome of SFTSV infection.

Variable	Assignment
Underlying condition	No = 0, Yes = 1
BMI	18.5～24 = 0, <18.5 or >24 = 1
Interval from illness onset to confirmation	<3 days = 0, 3 days- = 1, 6 days- = 2, 9 days- = 3
Chill	No = 0, Yes = 1
Headache	No = 0, Yes = 1
Fatigue	No = 0, Yes = 1
Muscular soreness	No = 0, Yes = 1
Conjunctival congestion	No = 0, Yes = 1
Petechiae	No = 0, Yes = 1
Gingival hemorrhage	No = 0, Yes = 1
Anorexia	No = 0, Yes = 1
Nausea	No = 0, Yes = 1
Vomiting	No = 0, Yes = 1
Haematemesis	No = 0, Yes = 1
Abdominal pain	No = 0, Yes = 1
Abdominal distension	No = 0, Yes = 1
Diarrhea	No = 0, Yes = 1
Lymphadenopathy	No = 0, Yes = 1

**Table 4:** Single variable analysis on factors for fatal outcome of SFTSV infection.

Variable	B	SE	Wals	P	OR	95% CI	
Underlying condition	1.074	0.552	3.782	0.052	2.927	0.992	8.639
BMI	1.362	0.549	6.152	0.013	3.903	1.331	11.449
Interval from illness onset to confirmation	0.691	0.276	6.273	0.012	1.996	1.162	3.428
Chill	−0.303	0.535	0.321	0.571	0.738	0.259	2.107
Headache	−0.887	0.551	2.593	0.107	0.412	0.140	1.212
Fatigue	0.282	0.798	0.125	0.724	1.326	0.277	6.343
Muscular soreness	0.244	0.550	0.196	0.658	1.276	0.434	3.751
Conjunctival congestion	0.522	0.586	0.793	0.373	1.686	0.534	5.319
Petechiae	0.379	0.626	0.366	0.545	1.460	0.428	4.980
Gingival hemorrhage	1.342	0.620	4.687	0.030	3.826	1.135	12.891
Anorexia	−0.316	0.550	0.329	0.566	0.729	0.248	2.143
Nausea	0.700	0.571	1.501	0.221	2.014	0.657	6.170
Vomiting	0.469	0.542	0.746	0.388	1.598	0.552	4.625
Haematemesis	0.935	0.865	1.170	0.279	2.548	0.468	13.869
Abdominal pain	−0.687	0.789	0.759	0.384	0.503	0.107	2.362
Diarrhea	0.739	0.540	1.871	0.171	2.093	0.726	6.030
Lymphadenopathy	−0.308	0.677	0.206	0.650	0.735	0.195	2.773
